# [Retracted] Isorhamnetin inhibits IL-1β-induced expression of inflammatory mediators in human chondrocytes

**DOI:** 10.3892/mmr.2025.13595

**Published:** 2025-06-17

**Authors:** Jin Li, Ruishan Wu, Xiaoping Qin, Dongyang Liu, Fenjie Lin, Qinglu Feng

Mol Med Rep 16: 4253–4258, 2017; DOI: 10.3892/mmr.2017.7041

Following the publication of this paper, it was drawn to the Editor's attention by a concerned reader that certain of the western blot data shown in Fig. 3B on p. 4256 were strikingly similar to data that had already appeared in a paper written by different authors at different research institutes that had already been published in the journal *OncoTargets and Therapy*.

In view of the fact that the abovementioned data had already apparently been published prior to its submission to *Molecular Medicine Reports*, the Editor has decided that this paper should be retracted from the Journal. The authors were asked for an explanation to account for these concerns, but the Editorial Office did not receive a reply. The Editor apologizes to the readership for any inconvenience caused.

